# A Combination of Platelet-Rich Fibrin and Collagen Membranes for Sinus Membrane Repair: A Case Report (Repair of Sinus Membrane Perforation)

**DOI:** 10.3390/dj11030084

**Published:** 2023-03-17

**Authors:** Anass Koleilat, Alaa Mansour, Fatma M. Alkassimi, Alfredo Aguirre, Bandar Almaghrabi

**Affiliations:** 1 Private Practice, Stow, OH 44224, USA; 2 Periodontology Unit, College of Dentistry, Sharjah University, Sharjah P.O. Box 27272, United Arab Emirates; 3 Department of Basic & Clinical Oral Sciences, College of Dental Medicine, Umm Al Qura University, Makkah, KSA P.O. Box 715, Saudi Arabia; 4 Department of Oral and Maxillofacial Pathology, UB SUNY School of Dental Medicine, Buffalo, NY 14214, USA; 5 Department of Restorative Dentistry, UB SUNY School of Dental Medicine, Buffalo, NY 14214, USA

**Keywords:** sinus grafting, Schneiderian membrane, maxillary sinus membrane perforation, dental implant, tooth extraction, antral pseudocyst

## Abstract

Maxillary sinus lift surgery is applied to compensate for the reduced vertical height in the posterior maxilla to facilitate placing a dental implant of a suitable length. Pathological conditions may be accidentally discovered, which necessitate careful assessment and management to prevent the infection of the maxillofacial complex and eventually bone grafting and dental implant failure. This case report describes an approach for the management of Schneiderian membrane perforation associated with the removal of an antral pseudocyst for successful dental implant therapy. A 70-year-old healthy Caucasian male presented for implant therapy to replace a non-restorable maxillary molar. Initial examination revealed the need for a sinus lift procedure to prepare the site for implant placement. A 3D CBCT evaluation before surgery revealed an incidental finding of a pathological lesion at the surgical site. The histological analysis of a biopsy specimen retrieved during implant site preparation showed findings consistent with antral pseudocyst. The resulting perforation of the sinus membrane was treated, and an adequate period of healing was given. A thickened sinus membrane was detected upon surgical exposure for implant placement. The novel technique illustrated could result in a fibrotic repaired sinus membrane and help shorten the time required for dental implant treatment.

## 1. Introduction

Dental implant therapy has become a well-established treatment modality for replacing missing and non-restorable teeth in the anterior and posterior regions with high survival rates [1]. The success of dental implants is associated with the presence of adequate bone volume [2]. The factors that may limit implant placement in the posterior maxilla include reduced vertical height and sinus pneumatization due to postextraction bone resorption [3]. In such clinical situations, the maxillary sinus lifting procedure is usually suggested as the treatment option of choice for placing conventional dental implants, with an excellent survival rate [4,5,6]. The lateral window approach is the most commonly used technique for sinus floor augmentation when the residual vertical height is less than 5 mm [7]. Multiple types of bone graft materials (i.e., autogenous bone grafts, allografts, xenografts, and alloplasts) have been suggested for the augmentation of the maxillary sinus to sustain the lifted space [8]. The collapsing of the sinus graft should be taken into consideration in the long term; therefore, material selection and the extent of graft volume should be thoroughly studied [9]. Sinus graft stability depends on the graft material used with more reduction in graft height for demineralized bone allograft and more stability for xenograft. This is explained by the mineral content and type of osseous healing. Simultaneous sinus lifting and implant placement is another option, provided that implant stability is achieved for the implant to support the lifted membrane [10].

A healthy Schneiderian membrane is crucial for the successful integration of grafting materials and obtaining high survival rates for implants inserted into augmented sites [11]. Nonetheless, the perforation of the Schneiderian membrane is a common drawback of sinus lifting surgery with an incidence rate reaching up to 60% [12,13], which can be significantly reduced with the application of the piezoelectric technique [14]. If the perforation defect is left untreated, it may entail the development of further postoperative complications such as sinus infections, loss of bone graft material, and increased implant failure rate [15,16]. The management of a lacerated sinus membrane depends on the perforation size. Usually, for small to medium perforation defects (i.e., less than 10 mm), treatment can be attained by elevating more of the intact membrane so that it folds over itself; then, a resorbable collagen membrane is used to seal the defect site prior to bone grafting [17]. The adjunctive use of biologics has been recommended to stimulate angiogenesis and enhance new bone formation, which results in improved healing and a shortened recovery period [18,19]. In this regard, the application of platelet-rich fibrin (PRF) is gaining much attention, as it allows for in situ enrichment with a variety of growth factors essential for tissue healing [20]. Unlike platelet-rich plasma (PRP), PRF is obtained via centrifugation without anticoagulants, and the resulting fibrin matrix comprises a variety of bioactive molecules such as transforming growth factor-beta (TGF-β), the vascular endothelial growth factor (VEGF), and the platelet-derived growth factor (PDGF), which promote cell proliferation and differentiation [21]. Some studies claim the use of PRF alone as a valid treatment for sinus lift cases, with their results showing significant promotion of bone gain and excellent implant survival. Other studies reported the additional use of PRF with bone grafting materials, which helps reduce the healing time and the better handling of the graft material. Overall, it seems that several factors support the application of PRF for sinus lifting procedures including its ease of use, minimal cost, and high success rates [22]. On the other hand, large-size defects can be treated using a Loma Linda pouch or require additional stabilization with sutures or tacks [23]. With the increased surgical morbidity with larger sinus membrane perforations, implant replacement therapy should be modified into multiple stages to separate membrane repair and grafting procedures from implant placement [24,25].

The presence of cystic lesions in the sinus, such as antral pseudocysts, has been reported as a factor that could increase the risk of perforation during the sinus lifting operation [26,27]. Cone beam computed tomography (CBCT) has been recommended for preoperative evaluation, as it provides a three-dimensional (3D) image of the available bone volume in the posterior maxilla and helps assess the health or pathology of the maxillary sinus [28].

Sinus augmentation in patients with sinus cysts is still an issue of controversy. It has been suggested that their presence may contraindicate sinus grafting, which should be carried out at least 6 months after cyst removal [29]. Large perforation defects of the Schneiderian membrane are usually encountered during cyst removal, and therefore, a variety of techniques have been investigated to repair large sinus membrane perforations; however, no proven method has been proposed as the gold standard treatment option.

This case report describes a new technique that could be used for the management of a large sinus membrane perforation resulting from the enucleation of antral pseudocysts. In this approach, the perforation site is repaired using a combination of PRF and multiple layers of collagen material to ensure the proper sealing of the sinus membrane tear before implant placement.

## 2. Clinical Presentation

**Figure 1:**
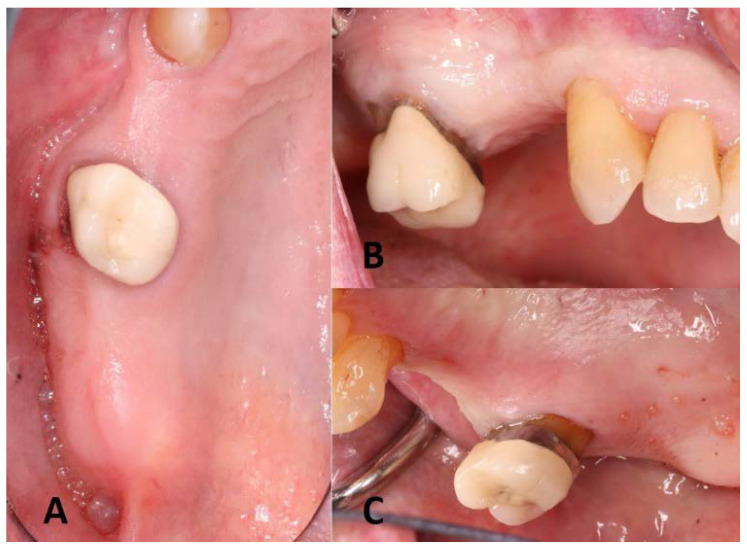
Preoperative clinical assessment: (**A**) occlusal; (**B**) buccal; (**C**) palatal view of the maxillary right sextant.

A 70-year-old male presented to the periodontics department at the school of dental medicine, the State University of New York at Buffalo, for the placement of dental implants in the edentulous area of the maxillary right molar area. The medical status was assessed, and no significant systemic diseases or conditions affecting the general health were reported. The intraoral examination revealed an edentulous area with adequate buccopalatal dimension on the maxillary right sextant. In addition, restorative consultation led to the clinical judgment of the non-restorability of the first molar (Figure 1).

**Figure 2:**
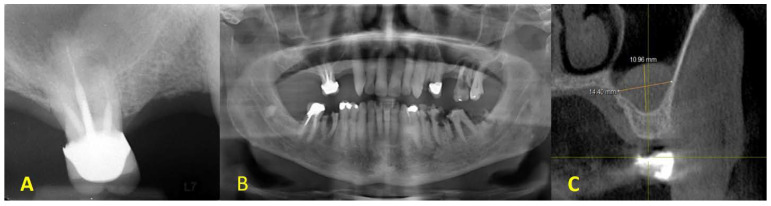
Preoperative radiographic evaluation: (**A**) homogenous solitary radiopaque mass observed on the right sinus floor in intraoral periapical with a periapical lesion on the MB root of the right maxillary molar; (**B**) a panoramic radiograph prior to initial therapy; (**C**) CBCT cross-sectional view of mucocele/antral pseudocyst with a patent osteomeatal complex.

Radiographic assessment using CBCT showed insufficient alveolar bone height for the placement of dental implants with adequate length on the posterior maxilla. Furthermore, a dome-shaped, radiopaque lesion was detected, and a differential diagnosis was proposed as mucocele or antral pseudocyst (Figure 2). After discussing the condition with the patient, the treatment plan was set as follows: (1) extraction of the first molar; (2) sinus membrane elevation in conjunction with the enucleation of the lesion detected to be sent afterward for pathological investigation; (3) sinus augmentation using a bone graft material; (4) a healing period of 6 months will be given before implant placement at the first premolar and first molar sites for an implant-supported bridge. Approval consent forms for the treatment plan and the surgical procedures were signed by the patient.

## 3. Surgical Procedures

All study procedures were performed according to the principles of the Declaration of Helsinki for Medical studies. A prophylactics antibiotic dose (i.e., 2 g of amoxicillin) was taken by the patient 1 h before the surgery [30]. The surgical procedures were performed under local anesthesia by giving 134 mg of articaine with 34 mcg of epinephrine (Septodont Septocaine^®^ articaine 4% HCL and epinephrine 1:100,000) as the posterior superior alveolar block and greater palatine block. Buccal and palatal infiltration using 34 mg of lidocaine with 36 mcg epinephrine (Xylocaine^®^ 2% with 1:50,000 epinephrine) was given to control the bleeding adjacent to the surgical site. The extraction of the first molar was completed by applying the least trauma possible to preserve the buccal bone. The evaluation of the extraction socket revealed a small perforation at the distobuccal root socket, whereas no perforation was noted on the mesiobuccal one. 

**Figure 3:**
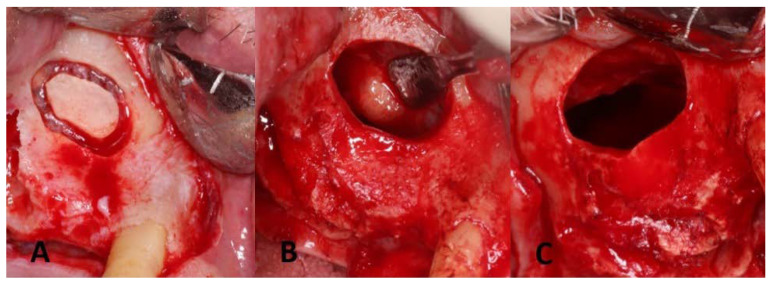
Lateral window preparation and the maxillary sinus cyst removal: (**A**) lateral window preparation with Piezo surgery; the bony island was detached and saved in saline; (**B**) sinus membrane after elevation and identification of the antral pseudocyst; (**C**) sinus membrane perforation after specimen removal.

**Figure 4:**
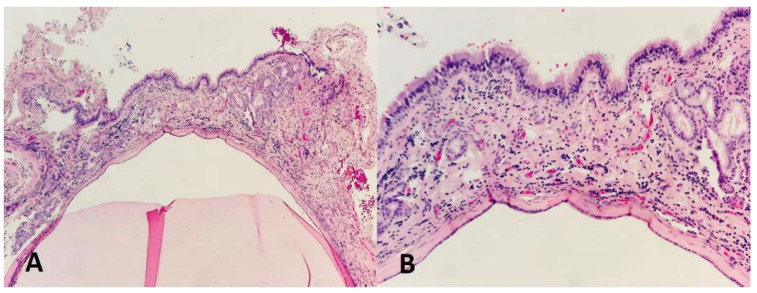
Histopathological assessment of the excised lesion: (**A**,**B**) showing pathological features of an antral pseudocyst with subepithelial inflammation that is composed of serum material and intermixed with inflammatory cells, covered by the sinus epithelium wall.

A midcrestal incision and two vertical releasing cuts were performed, followed by full-thickness flap reflection both buccally and palatally to expose the underlying bone. On the buccal aspect, the gingival flap was reflected to a level beyond the mucogingival junction to ensure adequate access to the maxillary sinus space. A lateral window was initiated about 5 mm from the crest, as seen in Figure 3A. A trapdoor technique was not performed because it would interfere with the access to the Schneiderian membrane for pathology assessment. Therefore, the bony island technique was selected instead. Upon achieving access to the sinus membrane, a yellowish nodule was noted and aspiration of the lesion revealed mucus-like material (Figure 3B). The removal of the lesion was achieved by gently peeling it off from the sinus membrane. A 15C blade with a sharp dissection of the sinus membrane was also used to help remove the lesion, which resulted in producing a large perforation in the Schneiderian membrane (Figure 3C). Pathological investigation was done to investigate the nature of the excised lesion which confirmed the diagnosis to be an antral pseudocyst (Figure 4). 

**Figure 5:**
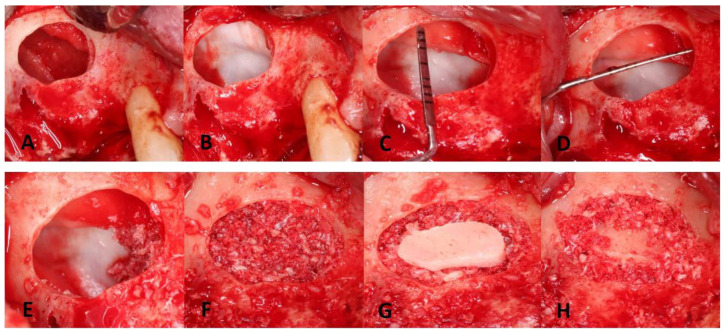
Membrane elevation with perforation repair and bone grafting: (**A**) sinus membrane elevation; (**B**) sinus repair with the first layer of CollaTape; (**C**) apico-occlusal dimension = 10 mm; (**D**) mesiodistal dimension = 10 mm with PRF membrane in place; (**E**) placement of a BioGide membrane to properly seal the perforation site. Note the bone graft on the anterior medial portion; (**F**) bone grafting of the surgical site well packed crestally; (**G**) the bony wall was placed over the lateral window, and then the crestal portion then the sinus cavity was filled; (**H**) bone graft around the bony island to stabilize the bony island.

Widening the osteotomy was carried out using a fine round diamond bur (Dentium Advanced Sinus Kit-DASK^®^, Cypress, CA, USA) at a speed of 1000 rpm. This facilitated further elevating the sinus membrane distally and posteriorly so that the membrane folded over itself to reduce the perforation size. Sinus perforation repair was then carried out, in agreement with the patient. For this purpose, two vials of the patient’s venous blood sample were first taken for preparing platelet-rich fibrin (PRF). The blood was placed in a 10 mL glass test tube, without anticoagulant, and then immediately centrifuged at a speed of 2700 rpm for 12 min [31]. To seal the lacerated Schneiderian membrane, a resorbable collagen material (i.e., CollaTape, ACE surgical supply, Brockton, MA, USA) was first applied, followed by the PRF membrane. The final step was to add one large layer of collagen membrane to ensure the proper sealing of the perforation (Bio-Guide, Geistlich Pharma, Wolhusen, Switzerland). Afterward, 5 ccs of cancellous bone (i.e., Allo-Oss, ACE surgical supply, Brockton, MA, USA) was well packed into the sinus providing enough height for future implants. Socket preservation at the first molar extraction site was also carried out. A ribose cross-linked collagen membrane (i.e., OSSIX PLUS™, DentSupply Sirona, Charlotte, CA, USA) was placed on the bony window, the ridge, and the extraction socket. A periosteal-releasing incision was performed to gain primary closure without tension. Laurel, horizontal mattress, and single interrupted sutures using 3-0 polytetrafluoroethylene (PTFE) sutures and 4-0 Glycolon were used to secure the flap over the surgical site (Figure 5). Post-op instructions including sinus precautions were conveyed to the patient. Additionally, pharmacological management included the following regimen:

Pseudoephedrine 30 mg for 3 days;

Amoxicillin 500 mg t.i.d. for 10 days [32];

Dexamethasone 0.5 mg t.i.d. for 6 days;

Ibuprofen 800 mg q.i.d. for 5 days.

Follow-up visits were scheduled at 10 days and 20 days postoperatively, and sutures were removed. Radiographs were taken during the healing period to make sure no sinus graft migration or sinus graft extravasation into the sinus cavity occurred. The patient did not report any symptoms of sinusitis, headaches, nasal obstruction, or infection.

**Figure 6:**
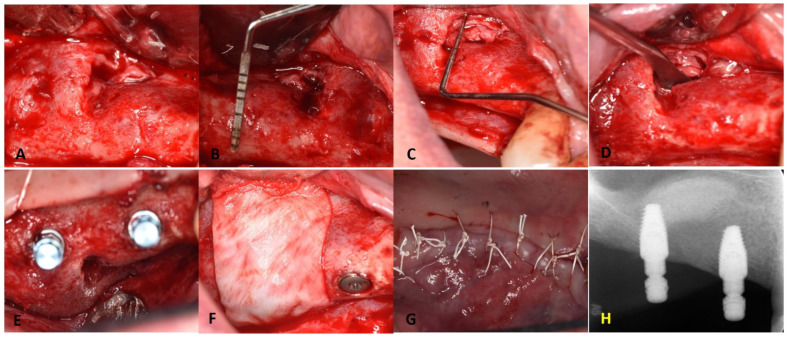
Surgical re-exposure for implant placement: (**A**) flap refection and exposure of previous sinus window; (**B**,**C**) adequate width and height for implant placement. Partial healing of the sinus window with evidence of fibrotic sinus membrane; (**D**) mini-sinus lift through the residual antrostomy; (**E**) implant placed in the prosthetic position; (**F**) collagen membrane placed over the antrostomy and crest; (**G**) achieving primary closure; (**H**) periapical radiographs for the dental implants placed at the right maxillary molar and 1st premolar sites with good parallelism, as seen by the implant carrier with additional bone graft into the sinus cavity, as revealed by the dome-shaped radio-opacity.

Six months postoperatively, a CBCT examination showed the presence of an adequate bone volume for implant placement. In brief, after reflecting a full-thickness flap, a partial closure of the lateral window with a thickened sinus membrane was revealed. A mini-sinus membrane lifting was performed combined with additional bone grafting. Osteotomy preparation was completed to place two 4.1 mm x 10 mm BLT implants (Straumann^®^ Implant), and implant angulation was verified with the aid of the patient’s occlusion using guiding pins and depth gauges. The procedures were completed with no complications encountered (Figure 6).

## 4. Discussion

The treatment plan was discussed with the patient regarding the replacement therapy on the maxillary posterior segment. Due to the insufficiency of the vertical bone height, a two-stage approach (i.e., maxillary sinus augmentation with delayed implant placement) was the treatment of choice [33]. Maxillary sinus grafting has proven to be a highly successful method and to give predictable results. Clinicians should be prepared to manage Schneiderian membrane perforation, which is the most common intraoperative complication of this procedure [34]. The increased risk of dental implant failure and bone graft infection is correlated to sinus membrane perforation [35,36]. However, the presence of maxillary sinus cysts, such as antral cysts, may increase the risk of such a complication, and therefore, it is speculated to be an absolute contraindication for sinus grafting procedures given the increased likelihood of graft infection with sinus membrane perforation [37]. Accordingly, this approach elongates the treatment duration required to finalize implant placement and might reduce patient satisfaction.

Other researchers suggested that sinus cysts removal is not required before or during a sinus augmentation operation. However, it has been illustrated that the cyst might enlarge, resulting in ostium obstruction and possibly increasing the risk of bone grafting and implant treatment failure [38]. It has been proposed that the aspiration and decompression of cysts during sinus lift surgery would be sufficient to reduce the size of the lesion and helps to decrease the internal pressure of the sinus, which also decreases the risk of perforation of the sinus membrane [39]. However, when there is an unclear diagnosis, enucleation should be considered [40]. Therefore, it is recommended to enucleate sinus cysts concurrently with sinus grafting to overcome such risks while taking into consideration that different management procedures are required to deal with the possibly increased size of sinus membrane perforation. The rationale for cyst enucleation before sinus augmentation includes ruling out malignancy, creating a healthier sinus environment, and lack of long-term data on the effect of pseudocysts on the graft material [37].

In our case, a large perforation occurred upon the removal of the sinus cyst, which is consistent with the observation from a previous report [27]. The use of autologous fibrin glue has been suggested for repairing sinus membrane perforation owing to its large content of platelets that release significant quantities of growth factors to promote wound healing [41]. However, further clinical trials are still required to validate its efficacy. Previous studies demonstrated the repair of such a defect with the sole application of a collagen sponge [25] or PRF [19,27]. Additionally, the combined use of the PRF and collagen membrane is shown to be efficient for sealing large sinus membrane perforation and enabling bone formation for subsequent implant placement [42]. A report of case series compared the bone height at sites with perforated Schneiderian membrane repaired using the PRF/collagen combination to others with intact membrane. The outcomes revealed a comparable increase in bone height in both situations at 6 months postoperatively, which could be attributed to the adhesive properties of the PRF membrane will act as a bridge between the edges of the perforation while applying the collagen membrane over the PRF will ensure the regeneration of the Schneiderian membrane [43].

The stability of the blood clot is essential during the initial phase of healing [44], which might be an issue with the fast degradation of the collagen sponge material or PRF affecting new bone formation [45]. Furthermore, it has been illustrated that in the case of large perforations, the use of a collagen membrane might not ensure adequate containment of the main material, which runs a risk of displacement when the graft material is placed [46]. We assume that our multiple coverage technique will allow achieving stability to enhance the healing outcomes.

In the current case, we further extended the repair process by using multiple layers of resorbable collagen membranes along with PRF to ensure the complete sealing of the large perforation resulting from the removal of the antral pseudocyst. PRF has multiple clinical benefits such as easy application procedure and good adaptation to the surgical site, in addition to the inclusion of several growth factors to support the healing process, especially for the large perforations of the maxillary sinus membrane [47]. The collagen materials applied can provide a scaffold for recruiting mesenchymal cells that help form connective tissue at the wound site [48]. Collectively, the combination of these materials could result in the formation of a thickened fibrous sinus membrane. Recently, the use of a biphasic scaffold enriched with autologous periosteal micrografts and a high percentage of progenitor cells has been introduced for sinus floor augmentation [49]. However, further clinical trials are recommended to validate its application in the case of Schneiderian membrane perforation.

Implant placement was delayed due to the risk of complications such as implant migration and acute infection. In addition, sinus graft material selection is important. In this regard, the use of a cancellous bone graft is favored due to its resorption rate [50], in case some bone particles migrated into the sinus. Even though the implant success rate is comparable regardless of whether implants are placed below the repaired or intact membranes, it seems that a higher failure rate exists with large perforations, and therefore, proper management is highly recommended [17].

In summary, the current case presents a novel and efficient treatment modality for a large maxillary sinus membrane perforation using multiple layers of collagen membrane material to ensure the proper sealing and containment of the graft particles for the subsequent implant replacement therapy. Additionally, we highlighted a group of key factors that should be carefully considered for the successful management of such cases, including proper radiographic interpretation, graft material selection, and good postoperative pharmacological management.

## 5. Conclusions

Membrane perforation is reported as the most frequent complication associated with maxillary sinus floor augmentation using the lateral window technique. The appropriate treatment planning is largely dependent on the actual size of the membrane perforation. The presented protocol appears to be a promising alternative approach for repairing a large sinus membrane perforation, which permits performing the repair process concurrently after maxillary sinus cyst aspiration and enucleation while ensuring the proper sealing of the sinus membrane perforation defect. However, more clinical trials with a larger sample size are advocated to validate the clinical results.

## Data Availability

No data were created.
